# Supplementary material for: Cloud BioLinux: pre-configured and on-demand bioinformatics computing for the genomics community
Source: BMC Bioinformatics. 2012 Mar 19;13:42. doi: 10.1186/1471-2105-13-42 (PMC3372431; doi:10.1186/1471-2105-13-42)
Supplement: Additional file 1 — Supplementary 1 Cloud BioLinux software documentation in the form of a mini, self-contained website. Users need to download and uncompress the .zip file, and open through a web browser the "index.html" file available on the main directory. (ZIP 1823 kb). [file 1471-2105-13-42-S1.ZIP › Cloud-BioLinux-Package-Documentation/docs/psa2msa.html]

Bio-Linux Software Documentation Pages

Back to search form

## psa2msa

|  |  |
| --- | --- |
| Name | psa2msa |
| Description | **psa2msa** is part of the pftools package, a collection of tools making use of generalised profiles for sequence analysis.  **psa2msa** reformats a **psa(5)** formatted profile-sequence alignment file into a Pearson/Fasta-formatted multiple sequence alignment file. *'psa file'* contains alignments of several sequence segments to the same profile in **psa(5)** format. |
| Homepage | http://www.isrec.isb-sib.ch/profile/profile.html |
| Remote Documentation | http://www.isrec.isb-sib.ch/ftp-server/pftools/pft2.3/documentation/psa2msa.1.html |
